# Cost Evaluation of Reproductive and Primary Health Care Mobile Service Delivery for Women in Two Rural Districts in South Africa

**DOI:** 10.1371/journal.pone.0119236

**Published:** 2015-03-09

**Authors:** Kathryn Schnippel, Naomi Lince-Deroche, Theo van den Handel, Seithati Molefi, Suann Bruce, Cynthia Firnhaber

**Affiliations:** 1 Health Economics and Epidemiology Research Office, Department of Internal Medicine, Faculty of Health Sciences, University of the Witwatersrand, Johannesburg, South Africa; 2 Right to Care, Johannesburg, South Africa; London School of Hygiene and Tropical Medicine, UNITED KINGDOM

## Abstract

**Background:**

Cervical cancer screening is a critical health service that is often unavailable to women in under-resourced settings. In order to expand access to this and other reproductive and primary health care services, a South African non-governmental organization established a van-based mobile clinic in two rural districts in South Africa. To inform policy and budgeting, we conducted a cost evaluation of this service delivery model.

**Methods:**

The evaluation was retrospective (October 2012–September 2013 for one district and April–September 2013 for the second district) and conducted from a provider cost perspective. Services evaluated included cervical cancer screening, HIV counselling and testing, syndromic management of sexually transmitted infections (STIs), breast exams, provision of condoms, contraceptives, and general health education. Fixed costs, including vehicle purchase and conversion, equipment, operating costs and mobile clinic staffing, were collected from program records and public sector pricing information. The number of women accessing different services was multiplied by ingredients-based variable costs, reflecting the consumables required. All costs are reported in 2013 USD.

**Results:**

Fixed costs accounted for most of the total annual costs of the mobile clinics (85% and 94% for the two districts); the largest contributor to annual fixed costs was staff salaries. Average costs per patient were driven by the total number of patients seen, at $46.09 and $76.03 for the two districts. Variable costs for Pap smears were higher than for other services provided, and some services, such as breast exams and STI and tuberculosis symptoms screening, had no marginal cost.

**Conclusions:**

Staffing costs are the largest component of providing mobile health services to rural communities. Yet, in remote areas where patient volumes do not exceed nursing staff capacity, incorporating multiple services within a cervical cancer screening program is an approach to potentially expand access to health care without added costs.

## Introduction

Millennium Development Goal number five, which calls for improvements in maternal mortality, was expanded in 2007 to explicitly include universal access to reproductive health services [1]. Reproductive health services address a broad range of issues such as maternal health, contraception, unintended pregnancy, safe and unsafe abortion, sexually transmitted infections (STIs), including HIV/AIDS, reproductive cancers, and sexuality generally [2]. However, many countries struggle to provide adequate access to even a limited package of services.

Access to healthcare has been described as having a number of dimensions: physical accessibility, financial affordability, and acceptability [3–5]. Physical accessibility is a major challenge globally for individuals in rural areas. For example, in South Africa, a nationally representative household survey showed that living in a rural area was a major factor contributing to poor access to health care [6]. Another study in South Africa found that households within a 30-minute walk were 10 times more likely to use the local clinic than households 90 to 120 minutes’ walk. Using mobile units, such as mobile vans, for delivery of health services is one option for addressing rural access.

Cervical cancer screening is a critical reproductive health service that is often unavailable to women in under-resourced settings. Cervical cancer is the fourth most common cancer in women globally; there were an estimated 528,000 cases diagnosed and 266,000 deaths from cervical cancer worldwide in 2012 [7]. In South Africa 30–40 per 100,000 cases are diagnosed each year as compared to less than 10 per 100,000 cases per year diagnosed in the United States [7]. The high incidence in the country is attributed to limited access to screening programs, a requirement for multiple clinic visits for women attending services, limited capacity in terms of skilled laboratory technicians, and limited time in busy clinics [8,9].

National statistics also mask acute problems in some areas and among certain populations. Reports from cervical cancer screening programs in HIV care and treatment settings in Johannesburg, South Africa indicate as many as 200 per 100,000 cases of cervical cancer diagnosed per year [10,11]. The HIV epidemic in South Africa plays a significant role in its cervical cancer incidence. The primary cause of cervical cancer is infection with human papillomavirus (HPV), and HIV infection in women has been shown to increase risks of HPV acquisition, persistent HPV infection, and progression to cancer [12–14]. Southern Africa bears the brunt of the global HIV epidemic; South Africa in particular has one of world’s largest domestic HIV epidemics with an estimated 6.1 million persons living with HIV and HIV prevalence of 17.9% for adults aged 15–49 [15]. Vaccines exist today which can prevent infection with HPV; however, roll out of these vaccines in South Africa has only just begun in early 2014 in a limited school-based program [16]. Although this program has the potential to make a difference in the very long term, it will not eliminate the need for cervical screening, nor does it address the problems of vulnerability to HPV infection by older, HIV-positive and negative women today.

The standard of care for cervical cancer screening in South Africa is the Pap test followed by referrals for colposcopy, histological diagnosis and treatment as needed [17]. However, access to and coverage of this screening service remain low; for 2010/2011, screening coverage for cervical cancer, defined as the proportion of women over 30 years of age who have received at least one Pap smear every 10 years, was estimated at 52% nationally [18].

**Fig 1:**
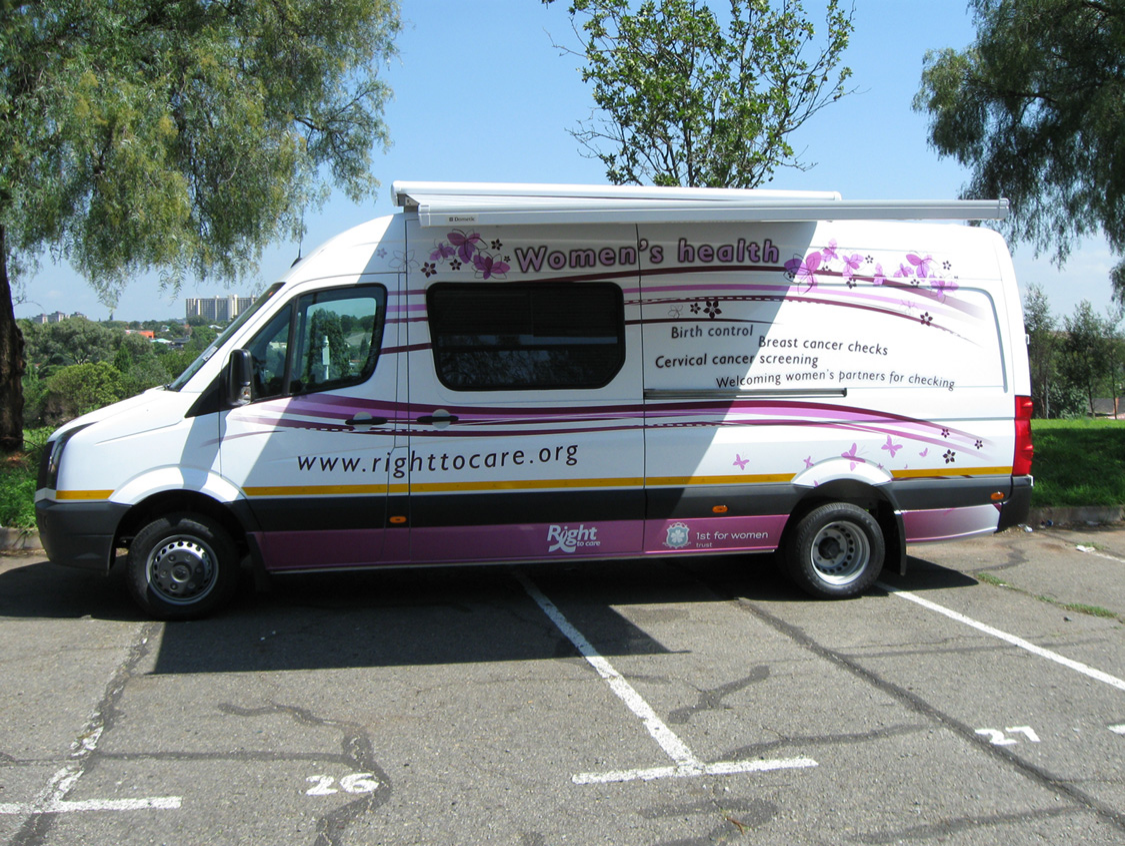
Women’s health van, South Africa. Photo by: Right to Care, 2015.

**Fig 2:**
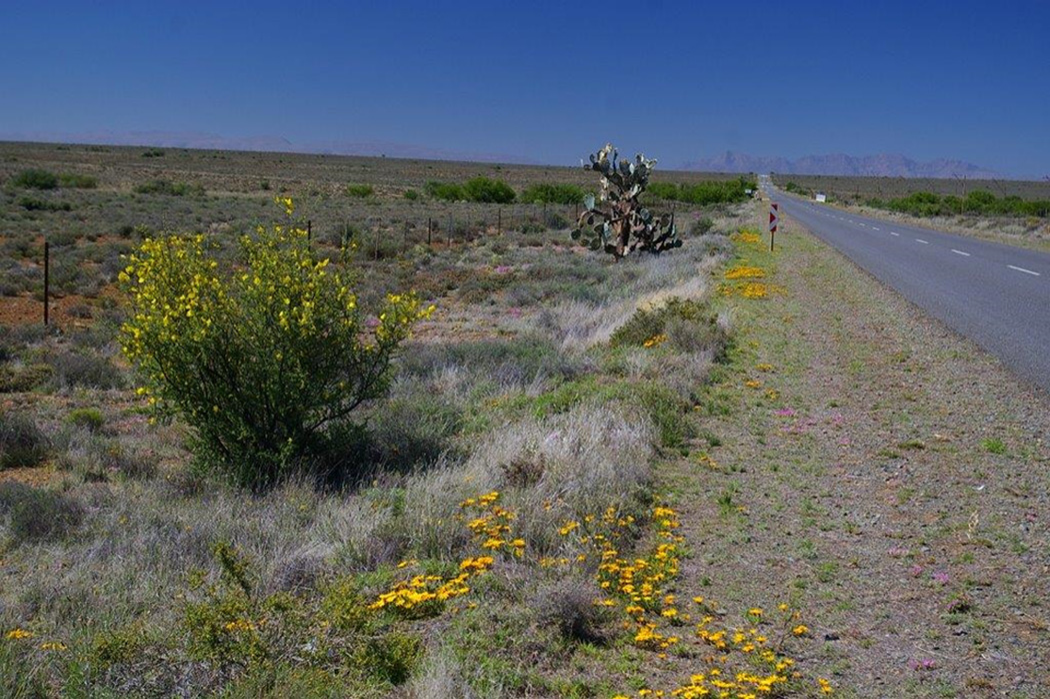
Sparsely populated Central Karoo district, South Africa. Photo by: Theo van den Handel, 2013.

Right to Care is a South African non-governmental organization that provides a range of health services and technical support for HIV/AIDS, tuberculosis (TB), STIs and cervical cancer through grants received from public and private sources. In 2013, cervical cancer screening services were provided annually by Right to Care to approximately 13,000 women across South Africa through various facilities. In November 2011, Right to Care started providing mobile van-based services in the Central Karoo district in Western Cape province (Fig. 1). An additional mobile unit was procured and started providing services in the Thabo Mofutsanyana District of the Free State province in April 2013. All areas of the Central Karoo are rural and sparsely populated (Fig. 2); whereas Thabo Mofutsanyana has a total population ten times that of the Central Karoo and multiple towns with populations of more than 50,000 persons [19]. Further characteristics of the two districts are in Table 1.

**Table 1:** Population and health indicators[19] for implementing districts.

	Thabo Mofutsanyana District	Central Karoo District	South Africa
Population	771,610	66,333	50,704,152
Population density (per km^2^)	27	2	42
Private hospitals/health facilities	2	0	216
Public primary health care clinics	64	8	3,146
HIV antenatal prevalence	30.7%	8.5%	30.2%

While mobile clinics are an integral part of the South African public health system (the Western Cape provincial government has 113 mobile clinics[20]), there have been few studies examining the costs of providing these mobile services [21,22]. In order to inform future budgeting and planning for the provision of these services, we conducted a descriptive cost evaluation of the two mobile clinics operating in these areas.

## Methods

### Program data

The primary target population for services offered via the mobile clinics is women; however, male partners and single men are also welcome. Although cervical cancer screening is a priority, both mobile units provide a range of health services (see Table 2 for a full listing). Patients are not charged for health services received. All clients who present at the mobile vans are offered a general medical history taking, blood pressure (BP) check, TB symptoms screening, and STI and candidiasis symptoms screening. Women are also offered a breast exam and cervical cancer screening, using a Pap test. The smears are collected and then sent to the National Health Laboratory System (NHLS) for review, according to the standard of care within the public health care sector. Results are sent both to the mobile unit and the local primary health care clinic; women with results requiring follow-up, including colposcopy, are referred to an appropriate public sector facility. Both units refer patients to local facilities if any general medical problems or TB symptoms are reported, or for cases of reported rape or other gender-based violence and for preventative services such as voluntary medical male circumcision.

**Table 2:** Services provided in mobile unit and number of women accessing each service, by district.

	Thabo Mofutsanyana District *	Central Karoo District
Evaluation period	1 Apr 2013–30 Sept 2013(6 months)	1 Oct 2012–30 Sept 2013(12 months)
Total number of women seen by mobile clinic^ǂ^	1,185	1,296
Pap smears taken	1,177	579
HIV counselling and testing provided	N/A	630
Contraceptive (injection or pills) dispensed	N/A	184
Male condoms (units dispensed)^^^	N/A	86,950
Female condoms (units dispensed)^^^	N/A	2,843
STI/candidiasis treated	N/A	116
Breast exam conducted	832	585

^ǂ^These individuals would have had a clinical history taken and received a BP check, TB and STI/candidiasis symptoms screen (see Methods).

^^^Boxes of condoms are also delivered to community sites.

* For the Thabo Mofutsanyana district, service volumes were multiplied by 2 to have 12-months for analysis.

The mobile unit in Thabo Mofutsanyana also refers individuals reporting STI/candidiasis symptoms as, at the time of the study, it was not equipped to provide the treatment required. In contrast, the mobile unit in the Central Karoo is able to provide medications for reported STI/candidiasis symptoms. This is done following South Africa’s syndromic management guidelines which prescribe treatment for “syndromes” rather than confirmed laboratory diagnosis of a condition [23]. The Central Karoo unit is also able to provide contraception when requested; however, as is the case in many of South Africa’s public facilities, this is limited to injectables and oral contraceptive pills (see Table 3 for specific types), and male and female condoms. The unit also distributes male and female condoms to other health facilities and distribution points. Finally, the Central Karoo unit also offers HIV counselling and testing (HCT). Individuals testing HIV-positive are referred for HIV staging, care and/or treatment to the nearest health facility offering these services.

**Table 3:** Variable^ǂ^ costs for services requiring consumables and/or medications.

Service	Item	Unit cost	Source
*Pap smears*			
	Brushes, slides, slide holders [in addition to kits supplied from laboratory]	$ 0.84	Program records
	Pap smear laboratory charge [inclusive of sample transport, supplies, results reporting]	$ 5.64	[28]
	Linen savers, gloves	$ 0.21	Program records
	Sterilizing liquid	$ 0.25	Program records
	*Average cost per patient*	*$6*.*94*	
*HIV rapid testing*			
	HIV rapid test kit	$ 0.51	[26]
	Testing consumables [alcohol wipe, safety lancet, plaster, cotton wool] per person	$ 0.40	Program records
	*Weighted average cost per patient (includes retesting for 10%)*	*$ 1*.*01*	
*Family planning*			
	Levonorgestrel /ethinyl estradiol [oral contraception, 28-day supply]	$ 0.22	[27]
	norethisterone enanthate [injectable contraception, 2 months]	$ 1.36	[27]
	medroxyprogesterone acetate [injectable contraception, 3 months]	$ 0.78	[27]
	*Weighted average cost per patient*	*$ 0*.*64*	
*Condoms*			
	Male condoms	$ 0.03	[25]
	Female condoms	$ 0.64	[25]
	*Weighted average cost per patient*	*$ 0*.*54*	
*STI/candidiasis treatment*			
	Cefixime [1 tablet, 400mg]	$ 2.33	Program records
	Erythromycin [20 tablets, 250mg]	$ 1.28	Program records
	Metronidazole [21 tablets, 200mg]	$ 0.24	Program records
	Ciprofloxacin [1 tablet, 500mg]	$ 0.55	Program records
	Clotrimazole [1 tube vaginal cream, 6 applicators, 50g]	$ 0.68	Program records
	Doxycycline [14 tablets, 100mg]	$ 0.30	Program records
	*Weighted average cost per patient*	*$1*.*63*	

^ǂ^ Variable costs are defined as costs that varied according to the number of patients seen and the type of service provided. Some services, e.g. medical history taking, blood pressure check, and symptom screening for TB and STIs, did not utilize medications or consumables and therefore have $0 variable cost. However, all services require expenditure on fixed costs (see Table 4).

Procurement and preparation of the vans to serve as mobile units was funded through a grant from a private local foundation. Medications (when dispensed) and consumables are procured by the provincial Departments of Health and distributed to the mobile units through public sector channels. Both vans employ a nurse and a driver who operate the mobile units four to five days per week using a variable route and schedule. The driver also assists as a lay counsellor for HCT and cervical cancer education. Both district programs keep daily statistics in terms of the number of patients visiting the mobile unit and the types of services provided (see Table 2 for patient volumes by service and by district); these numbers are reported to the funder and to the government’s district health information system. The programs also have vehicle logs recording kilometers travelled and program reports indicating communities served.

The CHEERS checklist was used in the preparation of this manuscript [24]. No patient-level data were evaluated for this costing study; a waiver was obtained from the Human Research Ethics Committee of the University of the Witwatersrand.

### Cost data

The analysis takes a provider perspective at the level of a facility (i.e. the mobile van). Costs incurred above the level of the facility, e.g. for district, provincial and national management of health care services, were excluded; only costs directly incurred for the mobile facility were included. Program service volumes were collected for the period 1 October 2012 to 30 September 2013 for the Central Karoo site and 1 April to 30 September 2013 for the Thabo Mofutsanyana site. Costs were collected through review of program expenditure records and/or national public sector pricing information [25–28]. All costs were collected in the local currency, the South African Rand, and inflated to 2013 costs using the South African Consumer Price Index [29] and converted to US dollars using an average exchange rate for the study period (9.27 South African Rand per US dollar) [30].

Variable costs, reported in Table 3, are defined as those costs which vary with patient volumes and the specific services provided. These include the medications and consumables required to provide the care on offer in each of the mobile vans. Ingredients-based costing, which relies on a standard “recipe” for service delivery, was used to determine the variable costs per service. For HCT and cervical cancer screening, ingredients-based costs were multiplied by reported service volumes. In addition, for HCT, the local testing algorithm indicates a repeat (i.e. confirmatory) test for all persons testing HIV-positive; thus an average positivity rate of 10% (based on program records) was assumed to create the weighted average cost of testing per person. To determine an average cost per women seen for STI/candidiasis treatment, because the medication dispensed varied based on the “syndrome” diagnosed by the nurse, detailed statistics kept for three months indicating treatment dispensed for STIs/candidiasis were used to construct weighted averages. These were then applied across all women noted to have received STI/candidiasis treatment for the evaluation period. For contraception, which also varied depending on the request, monthly statistics of the quantities of specific injectable and oral contraceptives dispensed were available for the entire study period and were used to create a weighted average per patient cost for these contraceptive methods. Finally, for condoms, no patient-level statistics are kept. Therefore, in line with general practice in public facilities, it was assumed that each patient who visited the vans received six male condoms and that 20% of women also received three female condoms. Then a weighted average cost for male and female condoms was calculated and applied across all patients reported to have received condoms.

Additional services provided which required use of equipment and staff time, but which did not consume medications or supplies were considered to have zero variable cost and therefore were excluded from Table 3. These included the general medical history taking, BP check, TB symptoms screening, STI and candidiasis symptoms screening, and breast exam.

Fixed costs, as per standard definitions for costing of health services [31], were defined as costs incurred regardless of the number of patients who utilized the mobile clinic. Fixed costs are reported as annualized figures in Table 4. These include equipment costs (e.g. the vehicle, fittings within the vehicle for cupboards and an examination couch, and medical equipment), vehicle and mobile clinic operating costs and staff. Staff was considered a fixed cost because the nurse and the driver were available at the mobile clinic regardless of the number of women accessing the clinic and regardless of the type of services the women received during their visit. Also, the staff contracts were a set cost for the time period (12 months) being analyzed here.

**Table 4:** Average annual fixed expenditures for both sites, in 2013 USD.

Type of expense	Item	Annual cost (2013 USD)^	Source
*Equipment* ^ǂ^			
	Medical equipment [set of metal specula, autoclave, baumanometer]	$ 204	Program records
	Panel van	$ 9,378	Program records
	Vehicle maintenance plan	$ 644	Program records
	Outfitting of van as mobile clinic	$ 3,693	Program records
	Diesel generator	$ 405	Program records
	Gas heater + gas cylinder	$ 361	Program records
	*Sub-total equipment costs*	*$ 14*,*685(16%)*	
*Vehicle operating costs*			
	Tires and minor repairs	$ 1,606	Program records,[34]
	Petrol, fuel	$ 5,482	Program records, [35]
	Vehicle insurance, licensing	$ 1,721	Program records
	*Sub-total vehicle operating costs*	*$8*,*822 (9%)*	
*Clinic operating costs*			
	Hand wash, towels, cleaning supplies	$ 1,002	Program records
	Office supplies	$ 1,693	Program records
	Medical waste management	$ 163	Program records
	Gas for heater	$ 233	Program records
	Diesel for generator	$ 1,779	Program records, [36]
	Communication, data	$ 432	Program records
	*Sub-total clinic operating costs*	*$5*,*302 (6%)*	
*Staff costs* *			
	Primary health care nurse	$ 50,361	[33]
	Driver/lay counsellor	$ 13,728	Program records
	*Sub-total staff costs*	*$64*,*089 (69%)*	
**Total annual fixed costs**		**$92,898**	

^ǂ^ Total costs for equipment are discounted at 5% per annum and annualized over 5 years as described in Methods. If no discount was applied (0% per annum), the total would have been $12,715 or $1,970 less.

^ For the Thabo Mofutsanyana district, fixed costs were adjusted from 6 to 12 months to allow for presentation of annual figures.

* Staff costs did not vary over the 12 months analyzed and did not vary according to the number of patients seen or the types of services provided and thus are included as fixed costs.

Annualized costs were calculated for equipment using an annual discount rate of 5% for consistency with other health economics evaluations [31]; 5% was also the average repo rate for South Africa in 2013 [32], over an assumed five years of use. Fixed operating costs were calculated as an average cost per year based upon expenditure records showing resource utilization for the program. The nurse and driver operating the vehicles are employed by Right to Care; salaries at Right to Care are aligned to public sector salary scales for health care workers. For this reason and for comparability to public sector services, public sector salary scales [33] were applied for the nursing staff in this analysis. All salaries are reported inclusive of benefits (e.g. pension, medical insurance, etc.) and are reported as total cost to employer.

Total program costs per year were calculated as the sum of the fixed and variable costs for each district. An average cost per patient seen was calculated by dividing the total costs by the total number of patients who received one or more services from each of the mobile units. The six months of patient statistics for Thabo Mofutsanyana were multiplied by two in order to create an estimated annual total.

Patient costs for accessing services were not collected, and are thus excluded from this analysis. In addition, the services provided in the vans do not require any additional training above the standard certifications or qualifications for the positions; therefore, no training costs are included.

## Results

As shown in Table 3, the variable cost and also the marginal cost of providing an additional Pap smear was $6.94 in both districts. Eighty-one percent of the variable cost for Pap smears was the NHLS laboratory charge, which included the cytology, transport of specimen, and standard consumables for specimen collection. For the mobile clinic in the Central Karoo, which offers a range of SRH services, Pap smears were the most expensive service provided. The variable costs of HCT averaged $1.01 per person tested including repeat rapid tests for persons with reactive initial tests, as per national testing algorithms. The variable cost of managing STIs/candidiasis for patients was the cost of treatment provided, the weighted average of which was $1.63 per patient. For contraceptive and condom provision, the marginal costs (again based on a weighted average cost) were $0.64 and $0.54 respectively.

Annualized fixed costs, shown in Tables 4 and 5, included establishing the equipped mobile clinic, clinic operating costs, and staff salaries for the two mobile units. These costs were the same at both sites. Kilometers travelled varied per month, but they were similar when comparing both districts. An average monthly distance travelled (2,366 km) was calculated incorporating the 18 months of data available from both districts (i.e. 12 months from the Central Karoo and six months from Thabo Mofutsanyana) and applied to both sites. As a result, as shown in Table 4, the average annual fixed costs for the van-based services totaled $92,898 at both sites. Annual cost-to-employer salaries ($64,089) for the nurse and driver/lay counsellor accounted for 69% of the overall annual fixed costs.

**Table 5:** Average annual total costs (fixed costs plus variable costs) and average per patient costs, in 2013 USD.

Per annum	Thabo Mofutsanyana District^ǂ^	Central Karoo District
*Annual fixed costs*	*$ 92*,*898*	*$92*,*898*
*Annual variable costs*	*$ 16*,*337*	*$ 5*,*639*
Pap smears taken	$16,337	$ 4,018
HIV counselling and testing provided	N/A	$ 636
Contraceptive (injection or pills) dispensed	N/A	$ 118
Condoms	N/A	$ 678
STI/candidiasis treated	N/A	$ 189
History taking, BP check, STI and TB symptom screening and breast exam^	$0	$ 0
**Total annual costs**	**$109,235**	**$98,537**
Average per patient cost	$46.09	$76.03

^ǂ^ Service volumes and fixed and variables costs were adjusted from 6 to 12 months to allow for presentation of annual figures.

^ Variable costs are defined as costs that varied according to the number of patients seen and the type of service provided. Some services, e.g. medical history taking, blood pressure check, and symptom screening for TB and STIs, did not utilize medications or consumables and therefore have $0 variable cost. However, all services require expenditure on fixed costs.

Table 5 provides total annual costs (fixed and variable) and average per patient costs for each district separately. Patient volumes and services offered were different for each of the districts, and thus the total annual cost and total variable cost varied by district. Variable costs for the year totaled $16,337 for the Thabo Mofutsanyana district, and $5,639 for the Central Karoo. In the Thabo Mofutsanyana district, the total variable costs for the year was equal to the costs of providing cervical cancer screening services; whereas in the Central Karoo, provision of cervical cancer screening comprised 71% of the total variable costs.

Considering both fixed and variable costs, the annual cost of providing mobile services in Thabo Mofutsanyana was $109,235, and the average annual cost for each of the estimated 2,370 women who might access the service over a 12-month period would be $46.09. In contrast, the annual cost of providing mobile services in the Central Karoo was $98,537, and the average cost for each person who utilized the service (n = 1,296) was $76.03. Although many of the patients received multiple services from the Central Karoo mobile clinic, the average cost per patient was higher because of the overall smaller number of patients seen. Fixed costs accounted for 94% and 85% of the annual cost of the mobile services programs in the Central Karoo and Thabo Mofutsanyana, respectively.

## Discussion

Fixed costs accounted for a significant proportion of the total annual costs of providing cervical cancer screening and other SRH and primary health care services via a mobile clinic in two rural districts, and the largest contributor to annual fixed costs was staff salaries. Thus, the cost of ensuring that rural communities have access to public health services through mobile clinics is largely driven by the cost of having a nurse and lay counsellor/driver available to provide these services. As this study was limited to program statistics and did not access patient-level records, further assessment of how staff time was spent was not possible. Future research into allocation of staff time across services may be useful in improving efficiency, given the high proportion of total costs for staff.

Fixed costs for both districts were the same as equipment can be procured centrally and distributed to remote areas without production capacity and as salaries for health care workers are also set centrally. Thus, the fixed costs calculated here can be reasonably applied to other rural districts in South Africa. Low patient volumes in the Central Karoo led to a much higher total (fixed plus variable) cost per patient ($76.03 compared to $46.09 in Thabo Mofutsanyana); sparse population is also the reason that the Central Karoo district has the highest per capita primary health care expenditure by the South Africa government [19]. For per patient costs, district population density per square kilometer should be used to consider which district costs calculated here are more applicable.

The variable cost of providing a Pap smear was $6.94 in both districts; most of this cost was for the laboratory charge which included the collection supplies, test and transport of the specimen and results. Marginal costs for providing additional, integrated services to the women, such as provision of contraceptives, management of STIs, HCT and TB screening were minimal in comparison. In addition, offering medical history screening, BP checks, TB and STI/candidiasis symptoms screening and a breast exam required no additional costs as a result of no additional consumables or supplies being needed to provide these services. Thus, especially in remote areas where patient volumes do not exceed the capacity of the nurse offering services, incorporating multiple SRH and other primary care services within a program for cervical cancer screening is one way to potentially expand access to a broader range of services without added costs.

### Limitations

This was a cost analysis and as such did not include the outcomes or effectiveness of the programs described. Other limitations of the study include the shorter period for analysis of services in Thabo Mofutsanyana. Our decision to multiply the six-month patient volumes by two to obtain annual figures may have under- or overestimated the services provided. Further, the evaluation period for Thabo Mofutsanyana began as service delivery started there; analysis of the program records from the start-up period in the Central Karoo indicate that initial patient volumes were much lower than the patient volumes achieved currently.

## Conclusions

In under-resourced settings, such as rural South Africa, women’s access to cervical cancer screening and other reproductive health and primary care services is often limited. Mobile vans equipped as clinics are one possible solution for improving access in these settings. Having skilled staff, in this case a nurse and lay counsellor/driver, is essential for the success of the program. Although some services can be provided with no added cost, as a result of no consumables or medications being required, the staff’s time is not unlimited, and trade-offs regarding which services to offer and/or prioritize should take into account local need.
